# Carbon ion radiotherapy for 80 years or older patients with hepatocellular carcinoma

**DOI:** 10.1186/s12885-017-3724-4

**Published:** 2017-11-07

**Authors:** Shintaro Shiba, Takanori Abe, Kei Shibuya, Hiroyuki Katoh, Yoshinori Koyama, Hirohumi Shimada, Satoru Kakizaki, Ken Shirabe, Hiroyuki Kuwano, Tatsuya Ohno, Takashi Nakano

**Affiliations:** 1 Department of Radiation Oncology, Gunma University Graduate School of Medicine, 3-39-22 Syowa-machi, Maebashi, Gunma 371-8511 Japan; 2 Gunma University Heavy Ion Medical Center, Maebashi, Gunma Japan; 3 Department of Diagnostic Radiology, Shibukawa Medical Center, Shibukawa, Gunma Japan; 4 Department of Medicine and Molecular Science, Gunma University Graduate School of Medicine, Maebashi, Gunma Japan; 5 Department of Hepato-Biliary and Pancreatic surgery, Gunma University Graduate School of Medicine, Maebashi, Gunma Japan; 6 Department of Surgical Science, Gunma University Graduate School of Medicine, Maebashi, Gunma Japan

**Keywords:** Carbon-ion radiotherapy, Hepatocellular carcinoma, Elderly patients, Radiotherapy

## Abstract

**Background:**

To evaluate the safety and efficacy of carbon ion radiotherapy (C-ion RT) for 80 years or older patients with hepatocellular carcinoma (HCC).

**Methods:**

Eligibility criteria of this retrospective study were: 1) HCC confirmed by histology or typical hallmarks of HCC by imaging techniques of four-phase multidetector-row computed tomography or dynamic contrast-enhanced magnetic resonance imaging; 2) no intrahepatic metastasis or distant metastasis; 3) no findings suggesting direct infiltration of the gastrointestinal tract; 4) performance status ≤2 by Eastern Cooperative Oncology Group classification; and 5) Child-Pugh classification A or B. Patients received C-ion RT with 52.8 Gy (RBE) or 60.0 Gy (RBE) in four fractions for usual cases and 60.0 Gy (RBE) in 12 fractions for close-to-gastrointestinal tract cases. Toxicities were classified using the National Cancer Institute’s Common Terminology Criteria for Adverse Events (Version 4.0).

**Results:**

Between March 2011 and November 2015, 31 patients were treated. The median follow-up period of all patients was 23.2 months (range: 8.4–55.3 months). Median age at the time of registration of C-ion RT was 83 years (range: 80–95 years). Child-Pugh grade A and B were 27 patients and 4 patients, respectively.

The 2-year estimated overall survival, local control, and progression-free survival rates were 82.3%, 89.2%, and 51.3%, respectively. No patients had Grade 2 or higher acute toxicities (within 3 months after C-ion RT). One patient experienced progression in Child-Pugh classification from A to B within 3 months after C-ion RT. In late toxicities, Grade 3 encephalopathy was observed in 3 patients, and 2 improved with medication.

**Conclusions:**

C-ion RT was effective with minimal toxicities for 80 years or older patients with hepatocellular carcinoma.

**Trial registration:**

UMIN000020571: date of registration, 14 January 2016, retrospectively registered.

## Background

Hepatocellular carcinoma (HCC) is the sixth most common cancer and the third major cause of cancer-related death worldwide [1]. Most HCC patients have a background of chronic liver disease resulting from alcohol abuse, infection of hepatitis C virus or hepatitis B virus [2, 3]. According to a 2005 report, the peak age range of HCC worldwide was 30 to 50 years [4]. On the other hand, in Japan, until 1990 the majority of HCC deaths were below the age of 69, but 66% of patients with HCC were over 70 in 2006 [3]. In light of the increased age of HCC patients, there is an urgent need for less-invasive local treatments.

Surgical resection, local ablation therapies including percutaneous radiofrequency ablation (RFA), and percutaneous ethanol infusion (PEI) are potentially curative treatments [2, 5]. However, many patients are not amenable to surgery or local ablation therapy for medical or anatomic reasons. Recently, proton therapy and stereotactic body radiotherapy (SBRT) with X-rays have been applied for HCC as less-invasive procedures [6–9]. Also, carbon ion radiotherapy (C-ion RT) has been used for HCC because of its excellent dose localization property and higher relative biological effectiveness based on the characteristics of higher linear energy transfer beam [10, 11]. Although the theoretical benefit of C-ion RT would exist in elderly HCC patients with hepatic dysfunction. There was a lack of data on the clinical outcomes of C-ion RT for elderly patients with HCC. In the current study, we analyzed safety and efficacy of C-ion RT in elderly HCC patients (80 years or older).

## Methods

### Patients

This retrospective analysis was performed using the medical records of patients treated with C-ion RT for eighty years or older patients with HCC at Gunma University Heavy Ion Medical Center (GHMC) between March 2011 and November 2015. All patients were treated and monitored according to the protocol approved by the Institutional Review Board. Eligibility criteria were: 1) HCC confirmed by histology or typical hallmarks of HCC using imaging techniques of four-phase multidetector-row computed tomography (CT) or dynamic contrast-enhanced magnetic resonance imaging (MRI) (hypervascular in arterial phase with washout in portal venous or delayed phases); 2) no intrahepatic metastasis or distant metastasis; 3) no findings suggesting direct infiltration of the gastrointestinal tract; 4) performance status (PS) ≤ 2 by Eastern Cooperative Oncology Group classification; and 5) Child-Pugh classification A or B. The disease stage according to the Union for International Cancer Control (UICC) classification (7th edition) [12] and The Barcelona Clinic Liver Cancer (BCLC) classification [13] were determined by CT, MRI, ultrasonography, and other variables. The model for end-Stage liver disease (MELD) score was calculated for evaluation of liver function in all patients [14]. HCC located within 2 cm of the main portal vein was defined as a porta hepatis group. In the current study, the uncontrolled tumors by transarterial chemoembolization (TACE) and/or transarterial infusion (TAI) and/or RFA were included. The treatment protocol for the current study was reviewed and approved by Gunma university Institutional Review Board, and all patients signed an informed consent form before the initiation of therapy.

### Carbon ion radiotherapy

Carbon ion beams were generated by synchrotron at GHMC. Passive scattering technique was applied for the treatment of HCC. Beam energies of 290 MeV/u, 380 MeV/u, and 400 MeV/u were employed. Beam energy was chosen according to the depth of the tumor. At our facility, XiO-N is used for treatment planning, which is XiO (Elekta)-based software incorporating a dose engine for ion beam radiotherapy (K2dose) [15–19] developed by the National Institute of Radiological Sciences, Japan, with interfaces from Mitsubishi Electric. Patients received C-ion RT once daily, 4 days per week (Tuesday to Friday). Radiation dose calculated for the target volume and surrounding normal structures was expressed in Gy (RBE), which was defined as the physical dose multiplied by the relative biologic effectiveness (RBE) of carbon ions [20, 21].

### Treatment planning and target delineation

Tailor-made fixation cushions and thermoplastic shells were used for the immobilization of patients for acquiring treatment planning CT images. After immobilization, respiratory-gated CT and four-dimensional CT (4-D CT) images were acquired. Images from the expiratory phase were used for treatment planning. Contrast-enhanced CT images were taken simultaneously and merged with treatment planning CT to precisely delineate the gross tumor volume (GTV). The clinical target volume (CTV) margin, including microscopic disease progression, was added to GTV, with an additional 5 mm in all directions. The internal margin (IM) was added as the extent of tumor motion shown in 4-D CT images. The planning target volume (PTV) was defined as a summation of CTV, IM, and setup margin. For daily patient position matching, fiducial gold marker was inserted in the liver. In the cases that lipiodol had been used in previous treatment, lipiodol was used as a marker for position matching of C-ion RT. Matching of the position of the fiducial marker was confirmed every day with two-directional X-ray images taken immediately before treatment.

### Dose prescription and fractionation

**Fig. 1:**
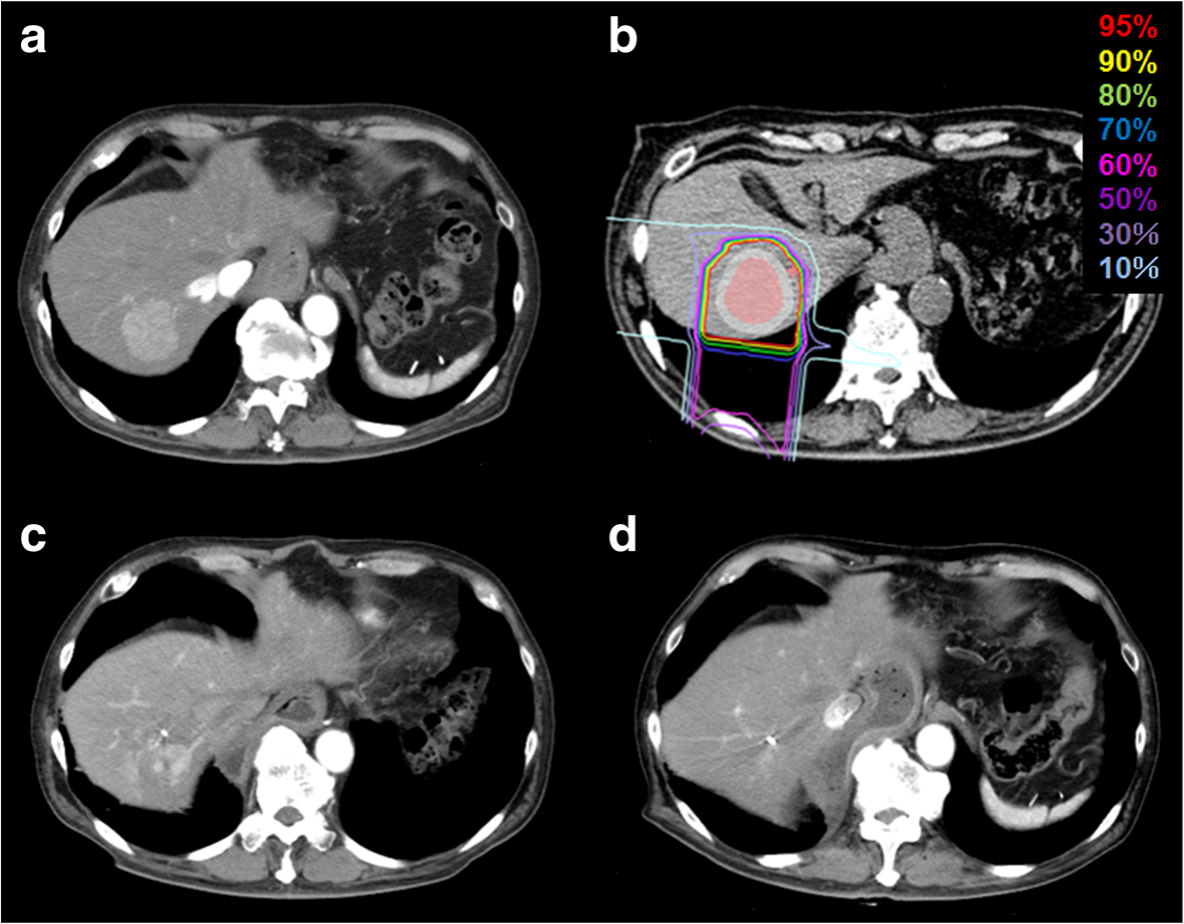
An 80-year-old male with HCC treated with C-ion RT. **a** CT before treatment. **b** Dose distribution on axial CT images. The area within the red outline is GTV and the area within the pink outline is CTV. **c** CT 3 months after treatment. **d** CT 18 months after treatment. There was a gastric tube reconstruction of esophageal cancer after surgery to the right of the vertebrae

Prescribed doses were 52.8 Gy (RBE) or 60.0 Gy (RBE) in four fractions for usual cases and 60.0 Gy (RBE) in 12 fractions for close-to-gastrointestinal-tract cases. The planning aim was to cover PTV with at least 90% of the prescribed dose. Dose constraints were: 1) D 1cm^3^ < 40 Gy (RBE) to the gastrointestinal tract; 2) V20 < 35% to the liver [22, 23]. The dose to the portal vein and bile duct was reduced as much as possible. Figure 1 shows a typical radiation field with dose distribution.

### Evaluation during follow-up

After completion of C-ion RT, patients were followed up one month after C-ion RT, and every 3 months thereafter. The examinations consisted of routine blood cell counts, blood chemistry and abdominal diagnostic imaging such as four-phase multidetector-row CT, dynamic contrast-enhanced MRI or contrast-enhanced ultrasonography. Acute and late toxicities were classified using the National Cancer Institute’s Common Terminology Criteria for Adverse Events, version 4.0 [24]. Acute toxicity was evaluated as the highest toxicity within 3 months from the initiation of C-ion RT. Late toxicity was evaluated as the highest toxicity after 3 months from the initiation of C-ion RT. Local recurrence was defined as tumor regrowth with enhancement of the contrast effect on CT or MRI or ultrasonography in the irradiated field with or without a continuous elevation of the tumor marker such as alpha-fetoprotein (AFP), *Lens culinaris* agglutinin-reactive fraction of AFP, and protein induced by vitamin K on blood test.

### Statistical analysis

Survival was measured from the date of initiation of treatment to the date of death or the most recent follow-up. Progression-free survival (PFS) was measured from the start of C-ion RT to the date of the first tumor progression disease or death from any cause. Probabilities of overall survival (OS), local control (LC) and PFS rates were calculated using the Kaplan-Meier method. The log-rank test was used to compare between 2 survival curves for univariate analyses. The Cox proportional hazard regression analysis was used to determine the implications of potential prognosticators. The statistical tests were two-sided, and a *p* < 0.05 was considered statistically significant. Factors with *p* < 0.1 in univariate analyses were included in the multivariate analyses. Variable risk was expressed as a hazard ratio with a corresponding 95% confidence interval. Wilcoxon signed ranks test was used for statistical analyses for difference in Child-Pugh score between before and 3 months after C-ion RT. All statistical analyses were performed using SPSS Statistics version 22 (SAS Institute, Tokyo, Japan).

## Results

### Patient characteristics

A total of 31 patients were treated with C-ion RT, and the patient characteristics are summarized in Table 1. Median follow-up of all patients was 23.2 months (range: 8.4–55.3 months). Median age at the time of registration for C-ion RT was 83 years (range: 80–95 years). Median tumor size was 45 mm (range: 15–93 mm). Prior treatment for the target region of C-ion RT was TACE in 10 patients, RFA with TACE in 2 patients and TAI in 1 patient. In patients with prior treatment, median duration between prior treatment and C-ion RT was 3.1 months (range: 1.6–13.2 months). There were no patients received a systemic therapy before C-ion RT. Seventeen of 31 patients were BCLC classification stage C. hree patients were due to portal vein invasion, 11 were due to performance status 1–2 and 3 patients were due to portal vein invasion and performance status 1–2. These patients were considered as indications for local treatments. In contrast, they were not to be indicated for systemic therapy and palliative therapy [25, 26].

**Table 1:** Patient characteristics (*n* = 31)

Characteristics	No.
Gender	
Male	22 (71%)
Female	9 (29%)
Performance status	
0	17 (55%)
1	12 (39%)
2	2 (6%)
Child-Pugh classification	
A	27 (87%)
B	4 (13%)
MELD score	
6–7	17 (55%)
8–9	11 (35%)
10 ≤	3 (10%)
Viral marker	
HBs-Ag (+), HCV-Ab (−)	2 (6%)
HBs-Ag (−), HCV-Ab (+)	18 (58%)
HBs-Ag (+), HCV-Ab (+)	1 (3%)
HBs-Ag (−), HCV-Ab (−)	10 (33%)
Co-morbidity	
Diabetes mellitus	10 (33%)
Hypertension	16 (52%)
Cardiovascular disease	8 (26%)
Respiratory disease	5 (16%)
Chronic renal failure	2 (6%)
Brain disease	2 (6%)
Tumor size, mm, median [range]	45 [15–93]
Serum AFP level, ng/ml, median [range]	7.3 [1.3–48,058.3]
Serum AFP-L3, %, median [range]	3.1 [< 0.5–84.2]
Serum PIVKA-II level, mAU/ml, median [range]	85 [10–19,937]
Stage (UICC 7th edition)	
I	24 (77%)
II	4 (14%)
III	3 (9%)
Stage (BCLC)	
A	13 (42%)
B	1 (3%)
C	17 (55%)

Abbreviations: *MELD* = Model for end-stage liver disease, *HBs-Ag* = Hepatitis B surface antigen, *HCV-Ab* = Hepatitis C virus antibody, *AFP* = Alpha-fetoprotein, *AFP-L3* = *Lens culinaris* agglutinin-reactive fraction of AFP, *PIVKA-II* = Protein induced by vitamin K, *AU* = Arbitrary unit, *UICC* = Union for International Cancer Control classification, *BCLC* = Barcelona Clinic Liver Classification

Three patients had 2 tumor lesions each, and they were contained within one CTV. Dose fractionation schedule was 52.8 Gy (RBE)/4 fractions in 15 patients, 60 Gy (RBE)/4 fractions in 9 patients, and 60 Gy (RBE)/12 fractions in 7 patients. When there were calculated as the biologically equivalent dose using an α/β ratio of 10 (BED10), C-ion RT of 52.8 Gy (RBE)/4 fractions, 60 Gy (RBE)/4 fractions and 60 Gy (RBE)/12 fractions were BED10 of 122.5 Gy, 150 Gy and 90 Gy. All patients completed C-ion RT as scheduled. Median hospital stay from the start of C-ion RT to discharge was 8 days (range: 5–23 days). Four patients were treated as outpatients.

### Overall survival and local control

**Fig. 2:**
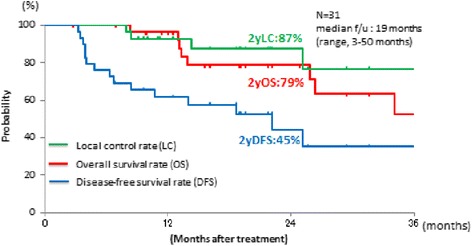
Overall survival, local control, and disease-free survival curves. Overall survival (*red line*), local control (*green line*), and disease-free survival (*blue line*) are shown for all patients treated with C-ion RT

The OS, LC and PFS curves of all patients are shown in Fig. 2. The 2-year estimated OS, LC and PFS rates were 82.3%, 89.2%, and 51.3%, respectively. At the time of analysis, 5 patients had died of HCC, and 3 died from intercurrent diseases (1 aspiration pneumonia, 1 pulmonary embolism, and 1 bile duct cancer). Fourteen of 31 patients were porta hepatis group. The 2-year OS and LC in porta hepatis group were 50.9% and 82.5%.

In the univariate analyses, there were significant differences for overall survival in performance status, and Child-Pugh classification (Table 2). In the multivariate analyses, there was significant difference for overall survival in performance status (Table 3).

**Table 2:** Local control and overall survival were analyzed by clinical characteristics (univariate analyses)

Factor	No.	2y–LC	*p*-value	2y–OS	*p*-value
Child-Pugh classification					
A	27	85.9%	0.544	85.0%	0.034
B	4	100%		37.5%	
Performance status					
0	17	90.9%	0.589	91.7%	0.015
1, 2	14	82.5%		64.6%	
MELD score					
6, 7	17	93.3%	0.705	81.8%	0.707
8 ≤	14	80.8%		75.5%	
Stage (UICC 7th edition)					
I	24	83.1%	0.866	81.3%	0.762
II, III	7	100%		71.4%	
Stage (BCLC)					
A, B	14	87.5%	0.498	100%	0.097
C	17	87.1%		67.2%	
Tumor size					
< 45 mm	14	90.0%	0.387	100%	0.540
45 mm ≤	17	86.2%		62.2%	

Abbreviations: *2y–LC* = 2-year local control rate, *2y–OS* = 2-year overall survival rate, *MELD* = Model for end-stage liver disease, *UICC* = Union for International Cancer Control classification, *BCLC* = Barcelona Clinic Liver Classification

**Table 3:** Overall survival was analyzed by clinical characteristics (multivariate analyses)

Factor	Hazard ratio (95% confidence interval)	*p*-value
Child-Pugh classification		
A vs B	4.937 (0.818–29.796)	0.082
Performance status		
0 vs 1 and 2	6.148 (1.189–31.807)	0.030
Stage (BCLC)		
A, B vs C	1.130 (0.277–4.605	0.864

### Toxicity

All of the observed acute and late toxicities are listed in Table 4. No patients had Grade 2 or higher acute toxicities. One patient experienced progression in Child-Pugh classification from A to B within 3 months. There was a no significant difference in Child-Pugh score between before and 3 months after C-ion RT (*p* = 0.803). As for late toxicities, Grade 3 encephalopathy was observed in 3 patients. One of these patients had chronic renal failure before C-ion RT. In this case, with the extensive progression of HCC and hepatic failure, encephalopathy did not improve. The other two Grade 3 encephalopathy cases improved to baseline before C-ion RT but both patients then developed intrahepatic metastasis. No patients had Grade 2 or higher other late toxicities such as dermatitis, pneumonitis, ascites, and rib fracture.

**Table 4:** Acute and late toxicities by CTCAE, version 4.0 (*n* = 31)

Acute toxicities					
Organs involved	G0	G1	G2	G3	G4
Dermatitis	2	29	0	0	0
Pneumonitis	23	8	0	0	0
Encephalopathy	30	1	0	0	0
Ascites	31	0	0	0	0
Late toxicities					
Organs involved	G0	G1	G2	G3	G4
Dermatitis	9	22	0	0	0
Pneumonitis	17	14	0	0	0
Encephalopathy	28	0	0	3	0
Ascites	26	5	0	0	0
Rib bone fracture	31	0	0	0	0
Change of Child-Pugh score after start of C-ion RT					
	≤ 0	+ 1	+ 2	+ 3	
Acute phase	26	4	1	0	
Late phase	29	5	1	0	

Abbreviations: *CTCAE* = Common Terminology Criteria for Adverse Events, *C-ion RT* = Carbon ion radiotherapy

In porta hepatis group, no patients had Grade 2 or higher acute toxicities. One patient experienced increasing 2 points of Child-Pugh score in acute and late phase.

## Discussion

With the increasing elderly population of HCC patients in Japan [3], a less-invasive and highly curable local treatment strategy has to be explored.

HCC has a number of local treatment options. Surgical resection is a well-established treatment, although its application has to be carefully selected in elderly patients. A meta-analysis by Huang et al. presented clinical outcomes of hepatectomy for HCC in 67 elderly patients (≥ 70 years old) and 268 control patients (< 70 years old) [27]. In their report, the 3-year OS and disease-free survival between the elderly and control groups were 55% and 40% (*p* = 0.017), and 58% and 41% (*p* = 0.157), respectively. The 2-year OS and disease-free survival rates, according to the Kaplan-Meier method, between elderly and control groups were approximately 65% and 50%, and approximately 60% and 52%, respectively. On the other hand, 9.0% of patients developed postoperative complications such as upper gastrointestinal hemorrhage and liver failure in the elderly group. Nozawa et al., in a report of surgical resection for HCC patients, divided patients into super-elderly (≥ 80 years old, *n* = 20), elderly (70–80 years old, *n* = 172) and younger (< 70 years old, *n* = 239) groups [28]. The 5-year OS in the super-elderly, elderly and younger groups were 67%, 60% and 65%, respectively. The 3-year tumor-free survival rates in the super-elderly, elderly and younger groups were 34%, 41% and 46%, respectively. The 2-year OS and 2-year tumor-free survival rates in the super-elderly group by Kaplan-Meier curves were approximately 100% and 10%, respectively. In these 2 studies, there was likely to exist a selection bias of the patients in the elderly groups, because inclusion criteria for resection was not fully described and because the elderly group generally presented favorable results compared with the control group. Nozawa et al. also reported a median postoperative hospital stay of 11 days in the super-elderly group [28]. Regarding complications, 30% of the super-elderly patients developed delirium ascribed to their long-term hospitalization, although psychiatric support and/or premedication were provided for the patients [28]. In addition, 10% of the patients developed cardiovascular disease and 5% of the patients developed abdominal infection and bile leakage. In the current study, the 2-year estimated OS and PFS were comparable with the result of surgery, although medically inoperable cases were included in our population. The median hospital stays with C-ion RT was 8 days, and 4 patients were treated safely as outpatients. No patients developed delirium or other severe complications, probably due to their short-term treatment period.

RFA and PEI are performed to treat unresectable small HCC. Tiong et al. reported a systemic review and meta-analysis of elderly patients with small HCC, 20–30 mm, treated with RFA and PEI [29]. The 3-year disease-free survival rates were 37–43% and 17–21%, respectively. Nishikawa et al. reported clinical outcomes of RFA for elderly (≥ 75 years) and control (< 75 years) patients with HCC of 20–30 mm [30]. They reported that the 3-year OS and recurrence-free survival rates between elderly and control groups were 64% and 84% (*p* = 0.001), and 21% and 40% (*p* = 0.001), respectively. The Kaplan-Meier method showed that the 2-year OS and recurrence-free survival rates between the elderly and control groups were approximately 75% and 90%, and approximately 35% and 55%, respectively. On the other hand, there was no significant difference in major adverse events related to RFA between the two groups (*p* = 0.670). In the current study, clinical outcomes were similar to those of HCC treated with RFA, despite the inclusion of larger tumors (median size, 45 mm). The indication for RFA is generally unresectable tumor of 30 mm or smaller, and is limited by anatomical situation. In contrast, C-ion RT can be applied for tumors larger than 30 mm or those anatomically untreatable with RFA [10].

Hata et al. reported proton therapy for 21 elderly patients (≥ 80 years old) with HCC [31]. Their 3-year OS and 3-year local progression-free rates were 62% and 100%, respectively, and no patient developed Grade 3 or higher toxicity except for thrombocytopenia in 2 patients. Their median fraction number was 22 (range: 10–35 fractions). In the current study, the number of fractions was 4 or 12, which was generally less than that of proton therapy. Together with the safety of C-ion RT, in the present study, C-ion RT also seems to be beneficial for elderly patients in terms of avoiding long-term hospitalization that can cause cognitive impairment.

There has been no analysis that focused on the outcome of elderly patients with SBRT with X-rays. Andolino et al. reported SBRT for HCC patients with a median age of 59 years and median tumor size of 31 mm [32]. Their 2-year OS and 2-year LC were 67% and 90%, respectively. There were no Grade 3 or higher acute non-hematologic toxicities. However, 20% of the patients experienced progression in Child-Pugh class within 3 months of treatment, 7 of 36 patients with Child-Pugh class A progressing to class B and 5 of 24 patients with class B progressing to class C. On the other hand, only one patient experienced progression in Child-Pugh classification from A to B within 3 months in our study. Abe et al. previously reported the results of dosimetric comparison between SBRT with X-rays and C-ion RT for HCC [22]. In their study, a low dose volume such as 5 Gy (RBE) to 20 Gy (RBE) for normal liver tissue was significantly lower with C-ion RT than SBRT. Therefore, compared with SBRT with X-rays, C-ion RT may have an advantage of conserving liver function.

Imada et al. reported comparison of efficacy and toxicity of C-ion RT for HCC located in the porta hepatis. They defined HCC located within 2 cm of the main portal vein as a porta hepatis group. They reported that the 3-year OS and LC in 18 patients were 44.4% and 87.8%. Acute adverse events of Grade 3 or higher were developed in 9 cases. As to Child-Pugh score in late phase, cases with changes in score increasing at least 2 points was five. In the current study, 14 of 31 patients has HCC located within 2 cm of the main portal vein as a porta hepatis group. The 2-year OS and LC in porta hepatis group were 50.9% and 82.5%. No patients had Grade 2 or higher acute toxicities. One patient experienced increasing 2 points of Child-Pugh score in acute and late phase. In the current study, clinical outcomes were comparable to results of Imada et al. reported, although the current study analyzed only elderly patients. Thus, this results suggested C-ion RT for elderly patients with HCC located in porta hepatis was effective and safe treatment.

There were some limitations to our study. First, this study was a single institutional retrospective analysis with a small number of patients and short follow-up duration. However, the follow-up period of this study was considered to be sufficient to confirm the safety because long-term radiation-related adverse events are uncommon except for radiation-induced malignancies. Second, the safety of C-ion RT for elderly HCC patients with Child-Pugh class B remained unclear due to small number of patients. Further investigation is necessary to confirm the safety of C-ion RT for elderly HCC patients with Child-Pugh class B. Third, clinical outcomes were not directly compared with other treatment modalities including surgical resection and local ablation therapies. However, the current study included not amenable to surgery or local ablation therapies cases due to the anatomical and medical reasons including poor PS and co-morbidity. This patient selection would not have contributed to the favorable results of this study.

## Conclusions

C-ion RT for patients 80 years or older with HCC was effective with minimal toxicities. This result suggested that C-ion RT may become an alternative treatment option for elderly HCC patients for whom surgery or local ablation therapies are not a viable choice. Further accumulation of clinical data with larger cohorts is warranted.

## Abbreviations

4-D CT: four-dimensional computed tomography
AFP: alpha-fetoprotein
AFP-L3: *Lens culinaris* agglutinin-reactive fraction of alpha-fetoprotein
AU: arbitary unit
BCLC: Barcelona Clinic Liver classification
C-ion RT: carbon ion radiotherapy
CT: computed tomography
CTCAE: Common Terminology Criteria for Adverse Events
CTV: clinical target volume
GHMC: Gunma University Heavy Ion Medical Center
GTV: gross tumor volume
HBs-Ag: hepatitis B surface antigen
HCC: hepatocellular carcinoma
HCV-Ab: hepatitis C virus antibody
IM: internal margin
LC: local control
MELD: Model for end-Stage liver disease
MRI: magnetic resonance imaging
OS: overall survival
PEI: percutaneous ethanol infusion
PFS: progression-free survival
PIVKA-II: protein induced by vitamin K
PS: performance status
PTV: planning target volume
RBE: relative biologic effectiveness
RFA: percutaneous radiofrequency ablation
SBRT: stereotactic body radiotherapy
TACE: transarterial chemoembolization
TAI: transarterial infusion
UICC: Union for International Cancer Control classification
